# Conservation features of the terrestrial Antarctic Peninsula

**DOI:** 10.1007/s13280-024-02009-4

**Published:** 2024-04-08

**Authors:** Jasmine R. Lee, Justine D. Shaw, Yan Ropert-Coudert, Aleks Terauds, Steven L. Chown

**Affiliations:** 1 School of Biological Sciences, Monash University, Melbourne, VIC 3800 Australia; 2 British Antarctic Survey, NERC, High Cross, Madingley Road, Cambridge, CB3 0ET UK; 3 Securing Antarctica’s Environmental Future, School of Biology and Environmental Science, Queensland University of Technology, Brisbane, QLD 4001 Australia; 4 Centre d’Etudes Biologiques de Chizé, UMR 7372, La Rochelle Université − CNRS, 79360 Villiers en Bois, France; 5 Integrated Digital East Antarctic Program, Australian Antarctic Division, Department of Climate Change, the Environment, Energy and Water, Kingston, TAS 7050 Australia; 6 Securing Antarctica’s Environmental Future, School of Biological Sciences, Monash University, Melbourne, VIC 3800 Australia

**Keywords:** Antarctica, Biodiversity, Conservation planning, Participatory process, Tourism, Values

## Abstract

Conserving landscapes used by multiple stakeholder groups requires understanding of what each stakeholder values. Here we employed a semi-structured, participatory approach to identify features of value in the terrestrial Antarctic Peninsula related to biodiversity, science and tourism. Stakeholders identified 115 features, ranging from Adélie penguin colonies to sites suitable for snowshoeing tourists. We split the features into seven broad categories: science, tourism, historic, biodiversity, geographic, habitat, and intrinsic features, finding that the biodiversity category contained the most features of any one category, while science stakeholders identified the most features of any stakeholder group. Stakeholders have overlapping interests in some features, particularly for seals and seabirds, indicating that thoughtful consideration of their inclusion in future management is required. Acknowledging the importance of tourism and other social features in Antarctica and ensuring their integration into conservation planning and assessment will increase the likelihood of implementing successful environmental management strategies into the future.

**Supplementary Information:**

The online version contains supplementary material available at 10.1007/s13280-024-02009-4.

## Introduction

The Antarctic Peninsula is changing rapidly. Increasing mean temperatures, recent record high temperature events (Robinson et al. 2020), growing glacial retreat (Wouters et al. 2015; Slater and Shepherd 2018), all time low sea ice extent (Fretwell et al. 2023), and predicted future climatic changes (Lee et al. 2017), suggest the direct and indirect impacts of climate change in the Antarctic Peninsula will be severe and ongoing (Siegert et al. 2019).

Simultaneously, human interest in the region continues to grow (Liggett et al. 2011; Hogg et al. 2020). As the most accessible and climatically mildest region of the continent, the Antarctic Peninsula hosts the largest proportion of scientific research stations (39 stations, 47% of all stations in Antarctica; COMNAP 2023), with regular proposals for new or expanded stations (e.g., Petrel Base on Dundee Island; Argentina 2022). Tourist numbers have also rapidly increased in the region since the early 1990s when only several thousand visitors visited the continent each year (Bender et al. 2016). The International Association of Antarctica Tour Operators (IAATO), the industry body coordinating Antarctic private sector travel (Haase et al. 2009), reports over 70 000 visitors landed on the Antarctic continent in the 2022/2023 season, with the vast majority concentrated at popular landing sites in the Antarctic Peninsula (IAATO 2023). Tourist activities are also diversifying. Kayaking, camping, snorkelling, snowshoeing and skiing are now common options on expedition cruises (Walton 2018; Netherlands and United Kingdom 2019). Although COVID-19 had a short-term impact on visitor numbers (Liggett et al. 2023), data from the most recent seasons suggest recovery is already complete and that numbers will continue to grow.

These growing pressures on the region have prompted rising concerns from Antarctic Treaty Consultative Parties (ATCPs), responsible for governance of the region south of 60° S (Antarctic Treaty Secretariat 1959), about the compound impacts of climate change and human activities, and the need for a more proactive approach to managing Antarctic tourism (New Zealand 2017; Netherlands and New Zealand 2019; Antarctic Treaty Secretariat 2023). Such concerns have led to an increased focus on the assessment of realised or likely impacts and their mitigation, and on prospective conservation planning approaches for the Peninsula region. Assessments have included, for example, trampling effects and methods to assess them (Tejedo et al. 2012), the effectiveness of visitor site guidelines (Cajiao et al. 2020), projections of likely invasive alien (non-native) species (Hughes et al. 2020), and a meta-analysis of the environmental impacts of tourism (Tejedo et al. 2022). The development of a systematic conservation plan for the Antarctic Peninsula was jointly proposed by the Scientific Committee on Antarctic Research (SCAR) and IAATO (Antarctic Treaty Secretariat 2017).

Systematic conservation planning (SCP) provides a diversity of options for identifying and prioritising protected area and/or management zone locations, in relation to goals that are identified jointly by stakeholders (Margules and Pressey 2000). The SCP process requires the identification of key features for a given spatial extent (or region) and the determination of the extent to which these are valued by stakeholder groups in that region (Knight et al. 2010; Guerrero and Wilson 2017). Stakeholder engagement is essential to ensure the effective implementation of evidence-based conservation recommendations (Knight et al. 2008, 2010; Ban et al. 2013). The engagement helps to tailor projects to suit stakeholder and policy requirements, generates acceptance of projects and outcomes, and encourages stakeholders to consider other’s needs more thoroughly (Knight et al. 2008; Reed 2008; Carwardine et al. 2019). It is also essential where stakeholders have expertise and knowledge that is not reflected in available quantitative data (Martin et al. 2012; Carwardine et al. 2019), a common challenge in Antarctica (Lee et al. 2022). Identifying features valued by each stakeholder enables policymakers to detect overlapping interests, thus pinpointing sites of common interest or potential conflict (sites or features that are valued by all stakeholders). Despite longstanding interest in the conservation of the Antarctic Peninsula (Lipps 1978; Bender et al. 2016), and accelerated interest in doing so given changing climate and increasing tourism (Siegert et al. 2019; Hogg et al. 2020), no explicit focus has been given to the identification of stakeholders and the features they value within the region.

Features identified by stakeholders as important during the SCP process generally reflect their core values. Traditionally, the values considered important in Antarctica, notably by the ATCPs, have mirrored the foundational values of the Antarctic Treaty (Hemmings 2012). These include preserving peace, maintaining the status quo on territorial claims, and protecting science and cooperation (Hemmings 2012). The 1991 Protocol on Environmental Protection to the Antarctic Treaty (hereafter the Environmental Protocol) ushered in recognition of a broader and more diverse set of values. Chief among these was acknowledgement that protecting the environment is as important as protecting peace and science (Hemmings 2012). Wilderness and aesthetic values were specifically recognised as intrinsic values requiring protection, alongside more typically recognised features of the Antarctic environment (Antarctic Treaty Secretariat 1991; Hemmings 2012; Summerson and Tin 2018). Article 3 2b of the Environmental Protocol clearly articulates that activities should avoid degradation of, or substantial risk to, areas of biological, scientific, historic, aesthetic or wilderness significance (reflected again in Annex V as reasons for designating an Antarctic Specially Protected Area; ASPA). Historic values were thus also recognised (Barr 2018). Although tourism has been the subject of discussion among the ATCPs at least since their fourth meeting in 1966 (Antarctic Treaty Secretariat 1966), and was specifically identified as an activity in the Environmental Protocol, tourism values and the features that reflect these were largely not considered. Yet identifying the values of all stakeholders and incorporating these into decision-making is essential for successful conservation and management of the region, especially to achieve broadly acceptable and hence effective systematic conservation planning outcomes (Bryan et al. 2011; Adams et al. 2019).

Here, we employ a semi-structured, participatory approach (Slocum 2005; Gill et al. 2008) to (i) identify features (or features of value) that are valued by stakeholders in the Antarctic Peninsula region, (ii) estimate overlap in stakeholder interest among features, and (iii) explore whether these features reflect the traditional science, environmental, and intrinsic values typically identified as core to the Antarctic Treaty System. This work forms the first component of the SCAR-IAATO systematic conservation planning project, a multi-year, interdisciplinary, multi-stakeholder project seeking to provide a tool to enable stakeholders to consider multiple options for addressing their diverse objectives within the region (SCAR and IAATO 2017, 2023). Identification of features is crucial for underpinning this SCP process. Once identified, conservation targets can be set for each feature, which are then used to prioritise protected areas and management zones across the region. Features in a SCP context generally represent key natural features used as surrogates for overall biodiversity in the planning and prioritisation process, such as species or vegetation types (Margules and Pressey 2000). They can also represent other spatially defined natural, social, or cultural values, such as carbon sequestration or development areas (Whitehead et al. 2014; Maxwell et al. 2020).

## Materials and methods

Aligned with the objectives of the SCAR-IAATO SCP project (SCAR and IAATO 2017, 2023), we characterised the terrestrial Antarctic Peninsula to contain features of value in three primary groups: biodiversity and the environment (hereafter biodiversity), science, and tourism. Stakeholders/experts were identified to represent those groups based on their primary interests. First, biodiversity was represented by Antarctic policymakers (the conservation of which is a primary aim of the Treaty Parties through the Environmental Protocol and its Annexes; Antarctic Treaty Secretariat 1991; Hughes et al. 2023), the Antarctic and Southern Ocean Coalition (ASOC; a coalition of non-governmental organisations working to conserve Antarctica and representing civil society), and life scientists (who work to understand and conserve biodiversity). Second, science was represented by Antarctic policymakers (where scientific research is accorded priority in the Antarctic Treaty Area through the Environmental Protocol; Antarctic Treaty Secretariat 1991) and Antarctic scientists (who contribute to organising research logistics and undertake the research in-situ). Third, tourism, the largest civil society activity in the region (e.g., Bender et al. 2016), was represented by IAATO operators.

We used a focus group approach that was semi-structured and participatory (Gill et al. 2008; Mukherjee et al. 2015) to identify features. The stakeholder elicitation process can be summarised in the following steps: (i) identification of experts to invite to participatory workshops (Slocum 2005; Mukherjee et al. 2015); (ii) pre-workshop preparation including provision of background information to participants (Gill et al. 2008); (iii) semi-structured focus group discussions (Gill et al. 2008) to identify stakeholder features, held during either in-person (tourism) or online workshops (science and biodiversity); (iv) broader discussion with stakeholders to aggregate and finalise lists of features identified during focus groups; (v) online surveys for participants to identify spatially-explicit sources of data for each feature; (vi) classification of features into categories and subcategories by the project team. Further details on each step are provided below and in Fig. S1.

### Pre-workshop preparation

Data collection occurred during and after targeted expert workshops in 2020. In-person workshops were planned for both the tourism experts, and science and biodiversity experts, but the science and biodiversity workshop were transitioned online due to the restrictions on travel imposed during the COVID-19 pandemic.

Prior to the workshops, participants received information introducing the project and the aims of the workshops (see Additional Online Material at: 10.26180/24023637). For the tourism workshop this consisted of a document outlining the aims of the broader systematic conservation planning project and the workshop goals, and a slideshow introducing the basics of systematic conservation planning. Further information and background were provided in the in-person and online workshops. To ensure that the online workshops involving science and biodiversity experts were efficient, video introductions to the project and introductions to features of value, as well as a proposed list of starting features, were provided in advance of the online workshops, alongside the introductory documents.

### Tourism workshop

The first workshop was held with tourism operators in Miami, USA in February 2020. All IAATO Operators were invited to attend the workshop. Twenty-six people participated, representing 17 IAATO expedition operators (~ 30% of all IAATO Operators; IAATO 2022). A further three yacht operators participated in the process remotely in May/June 2020. Most participants had worked in the Antarctic tourism industry for a substantial period (> 5 years, although some had > 20 years) as expedition leaders, guides, operations directors and/or senior managers.

The workshop began with an introduction to features of value and their use in conservation planning. Several pre-identified features (known to be visited by tourists; Lee 2019) were provided as examples, including pygoscelid penguin colonies and historic sites. Smaller focus groups (5–6 people) then identified and discussed features valued by the tourism industry in the Antarctic Peninsula. An IAATO staff member facilitated the discussions of each focus group and compiled notes on features identified. A member of each focus group presented their identified features to the wider group in the next session. The full group then approved or rejected each proposed feature based on the majority (i.e. whether or not it is valued by the industry at large). Workshop participants agreed on a total of 42 features important to the Antarctic tourism industry. Participants finally completed a survey identifying, in a spatially explicit manner, tourist landing sites that contained these features.

### Science and biodiversity workshop

The second workshop involved Antarctic science and biodiversity experts (primarily Antarctic scientists and policymakers) and occurred online using the e-meeting software Zoom (v 5.0.5) for focus group meetings, and the online discussion platform Slack (v 4.7) for further discussion and note taking. Participants were invited to take part in the workshops if they were a member of the SCAR–IAATO SCP liaison group or if they were identified for their specific expertise (e.g. seabird or invertebrate experts). A community liaison group was established in 2019 to provide input to the SCP process, which any Antarctic scientist or policymaker interested in the project could join. The liaison group was advertised to the SCAR community (via SCAR’s mailing list and website (www.scar.org) and the CEP (via its mailing list)). Fifty-five people participated in the online workshops (30 men, 25 women, from 17 different countries). All had substantial experience working in various fields of Antarctic science or policy and included experts on biodiversity, tourism, policy, conservation, and national program operations. Many participants had expertise in both science and biodiversity, and so identified features for both groups. Many of the scientists were life or social scientists, thus some science values from the physical sciences are likely to have been overlooked.

Participants took part in one of five online focus group discussions, which were scheduled at various times to cater for time-zone differences. The online focus groups followed the same format as the in-person tourism workshop, where an introduction to features and their use in conservation planning was followed by examples of features, then by focus groups and then a larger group discussion. The breakout feature of Zoom was used for the focus groups, which were facilitated by a member of the project team.

Despite the absence of key vertebrate and invertebrate groups from the terrestrial Peninsula (e.g. no amphibians or reptiles, and only two insect species), the region still has significant biodiversity at the species level (Chown et al. 2015). Unfortunately, most species are insufficiently well-known spatially (and in some instances taxonomically) for biodiversity features to be assessed at this level (Chown and Brooks 2019; Lee et al. 2022). Rather, 45 biodiversity groupings accepted as representative in a previous Antarctic Priority Threat Management assessment (Lee et al. 2022) were used as a starting point (see online material). Nonetheless, biodiversity experts identified several other biodiversity features based on their expert knowledge.

Following the online workshops, proposed features were combined into two lists (one for biodiversity features and one for science features). The two lists were then posted to Slack to allow workshop participants to see and discuss the features identified in the other discussion sessions. The lists were made available to participants for three weeks to partake in the online discussion and comment on the features. The lists of features were then finalised based on feedback from participants and a majority decision. In the case of a tie, e.g. whether ‘site accessibility’ is too vague to be considered a science feature, it was retained in the final list. Workshop participants agreed on a total of 72 features valuable for biodiversity, and 93 for science. Participants were then asked to identify sources of spatial data for each feature (where they were aware of such data) in an online survey.

### Feature classification

To assist in visualisation of the features, we grouped related features into seven major categories: biodiversity, geographic, habitat, historic, intrinsic, science, and tourism. The categories are generally broad and were based on the discussions from the focus groups. Within these categories, similar features were then further grouped together into subcategories. For example, Antarctic fur seals (*Arctocephalus gazella*), elephant seals (*Mirounga leonine*), and Weddell seals (*Leptonychotes weddellii*) were grouped into the ‘seal’ subcategory.

**Fig. 1:**
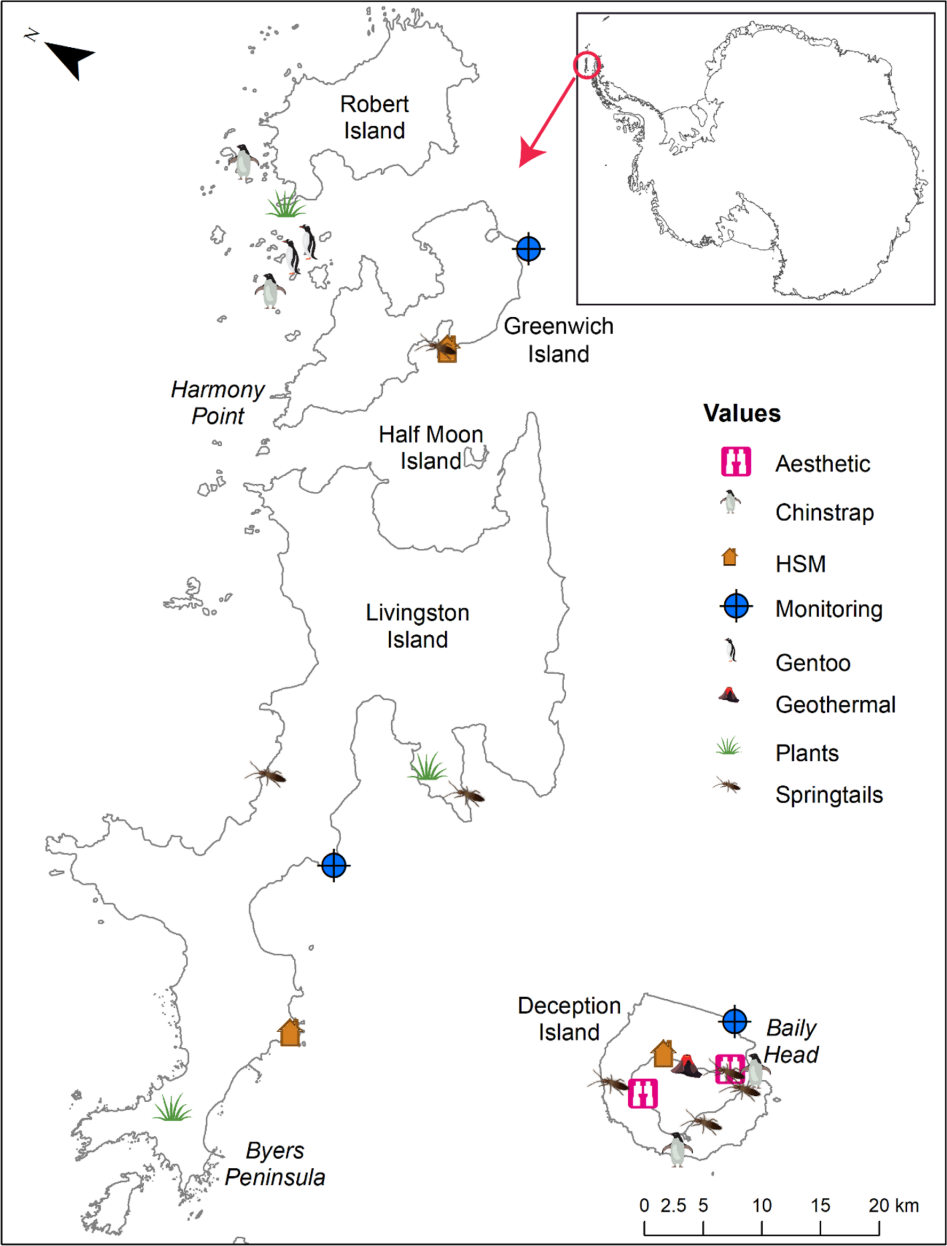
Map illustrating the spatial locations of some examples of the conservation features identified by stakeholders in the South Shetland Islands region of the Antarctic Peninsula. Inset shows Antarctica with a circle indicating the location of the South Shetland Islands

We also estimated the spatial data availability (data that identifies locations of features across the Antarctic Peninsula) for each feature using the expert information (see Fig. 1 for an example). We categorised these data availability as ‘comprehensive’, representing datasets with broad spatial coverage (often region wide) that were often quantitative; ‘ad hoc’, representing small datasets that only covered small areas and were often qualitative or anecdotal; or ‘not available’, where participants were unable to identify any known sources of spatial data.

Finally, given that Annex V of the Environmental Protocol provides the international framework for designation of Antarctic Specially Protected Areas (ASPAs), we also identified which features align with the outstanding values listed in Annex V. Examples include: ‘areas kept inviolate from human interference’ or ‘areas with important or unusual assemblages of species’ (see Table 1 for all values).

**Table 1:** Terrestrial conservation features in the Antarctic Peninsula that relate to the values identified in Annex V, Article 3 of the Environmental Protocol. This Annex recognises the purposes for which an area can be designated as an Antarctic Specially Protected Area (ASPA). Features are listed under the appropriate category recognised in Annex V. Full details for the features can be found in Table S1

Feature
**(a) areas kept inviolate from human interference so that future comparisons may be possible with localities that have been affected by human activities;**
New monitoring sites/control sites
Inviolate sites
**(b) representative examples of major terrestrial, including glacial and aquatic, ecosystems and marine ecosystems;**
Ecological processes
Microclimate diversity
Representative sites
**(c) areas with important or unusual assemblages of species, including major colonies of breeding native birds or mammals;**
Adélie penguins
Chinstrap penguins
Gentoo penguins
Emperor penguins
Skuas
Antarctic shag
Antarctic tern
Procellarids
Southern giant petrels
Kelp gulls
Greater sheathbill
Antarctic fur seals
Elephant seals
Barren soils
Cryoconites
Epi-shelf lakes
Fjords
Freshwater lakes, streams and meltwater
Nunataks
Geothermal/volcanic sites
Microbe hotspots
Newly exposed ice-free areas
Permafrost
**(d) the type locality or only known habitat of any species;**
Type localities *(not identified in the current surveys)^*
**(e) areas of particular interest to ongoing or planned scientific research;**
Non-native species sites
Important monitoring sites
Vulnerable sites
**(f) examples of outstanding geological, glaciological or geomorphological features;**
Glaciers
Fossil bearing rocks
Meteorites
Minerals and crystals
Geological processes
**(g) areas of outstanding aesthetic and wilderness value;**
Wilderness
Aesthetic values
**(h) sites or monuments of recognised historic value; and**
Historic sites and monuments (HSM’s)
Unofficial historic sites
**(i) such other areas as may be appropriate to protect the values set out in paragraph 1 above**
Potential expansion zones
Ecosystem services

^Type localities were not identified as a value by the workshop participants, though have been included in the table for comprehensiveness

R version 4.2.2 was used for data visualisation (R Core Team 2022).

## Results

**Fig. 2:**
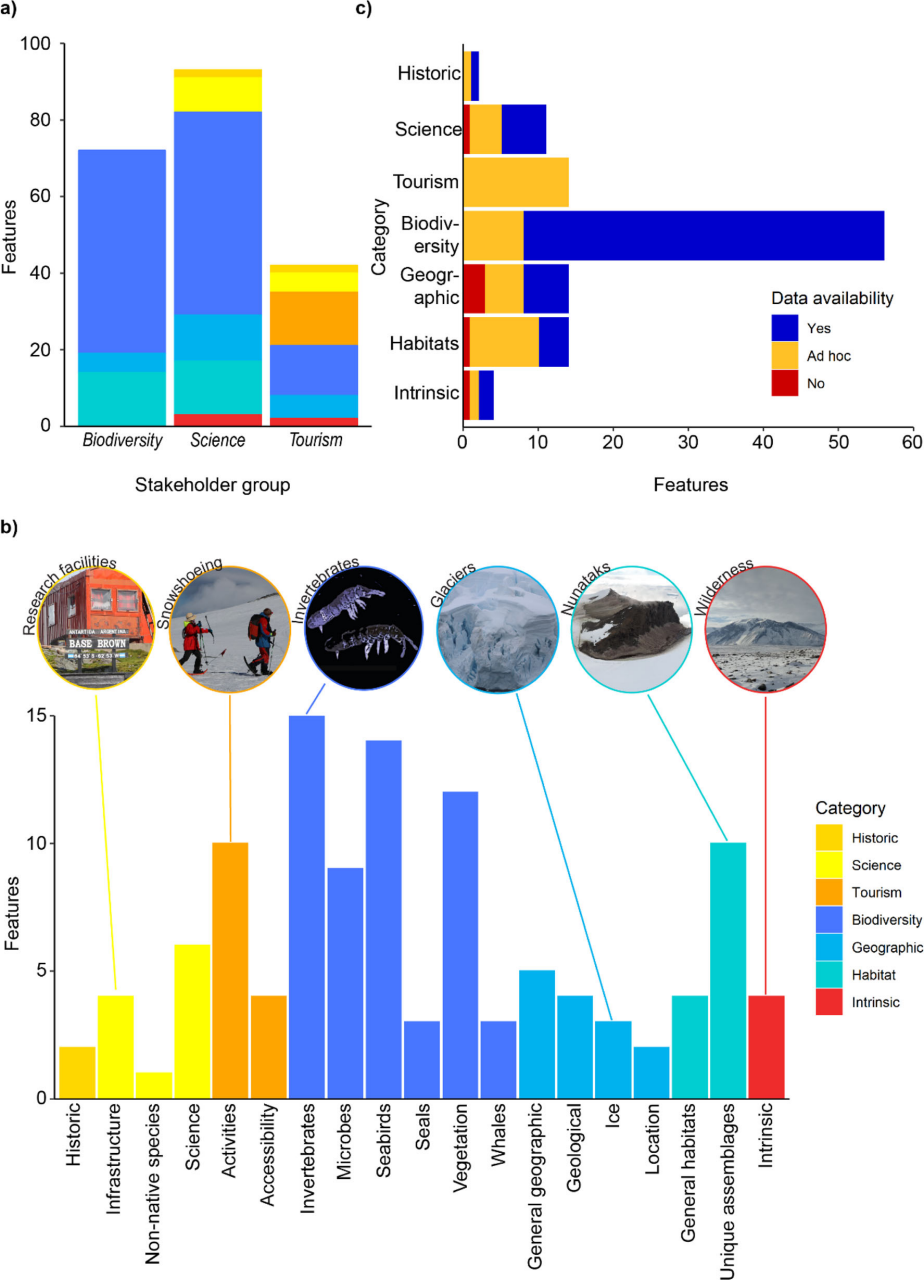
Terrestrial conservation features in the Antarctic Peninsula identified by stakeholders. **a** Number of features identified by each of three stakeholder groups, coloured by category. Some features were considered important by multiple stakeholders. **b** Number of features identified under 19 subcategories, that fall into seven broader categories (colours). Examples of features belonging to subcategories are identified in the pictures. **c** Number of features identified under each of the seven categories with colours indicating comprehensive data availability for those features. The numbers underlying this figure are found in Table S2.

Stakeholders identified a total of 115 features in the terrestrial Antarctic Peninsula (Table S1). Of these, tourism stakeholders identified 42 features of value, biodiversity stakeholders identified 72 features, and science stakeholders identified 93 features (Fig. 2a). Many features were identified as important by multiple stakeholder groups. The biodiversity features include taxonomic groups (e.g., crustose lichens) and other physical habitat features (e.g., geothermal sites). The science stakeholders included all of the biodiversity features in their list, given research undertaken on most of the assigned biodiversity features, as well as scientific infrastructure and other important geographic or scientific features, such as glaciers, fossil bearing rocks and important monitoring sites. Tourism stakeholders identified a diversity of features as being valuable, including some historic, biodiversity, geographic, and intrinsic features, alongside specific tourism features, such as camping sites.

With the 115 features split into categories, the largest category was biodiversity, with 56 features, followed by geographic, habitat and tourism with 14 each. The historic category contained the least, with only two features identified. Within subcategories (Fig. 2b), invertebrates contained the most with 15 features, followed by seabirds with 14 features, and vegetation with 12 features—reflecting the large overall number of biodiversity features identified, but also reflecting the initial division of species into taxonomic groups (following Lee et al. 2022).

Estimated spatial data availability for features varied substantially across categories (Fig. 2c). Data representing biodiversity features were estimated to be the most comprehensive, with every feature estimated to have some ad hoc data available and the majority to have comprehensive data available. Tourism features were estimated to have ad hoc data available representing every feature, though no comprehensive data were available for any. The other five categories have an approximately equal split of ad hoc data and comprehensive data available for features. Six features were estimated to have no spatial data available at all: soil sediments, ecological processes, microclimate diversity, crevasses, vulnerable sites, and ecosystem services.

Many features were identified as important by multiple stakeholder groups (Table 2; Table S1). At a category level, the biodiversity (53 out of 56), habitat (14 out of 14), and historic (2 out of 2) categories have the highest proportion of features valued by more than one stakeholder. The tourism category has the lowest levels of overlapping interest (0 out of 14), as all of the features were only identified as important by the tourism industry. Six features were identified as important by all three stakeholder groups, including the three pygoscelid penguins, emperor penguins (*Aptenodytes forsteri*), and elephant and fur seals.

**Table 2:** Number of features in each category that were identified as important by one or more stakeholders. Table S1 lists each feature and the stakeholders that identified it as important

Category	3 stakeholders	2 stakeholders	1 stakeholder	Total # of features
Biodiversity	6	47	3	56
Habitats	0	14	0	14
Geographic	0	9	5	14
Intrinsic	0	1	3	4
Historic	0	2	0	2
Science	0	3	8	11
Tourism	0	0	14	14
Total	6	76	33	115

Forty-two of the features identified by the stakeholders relate to the description of the outstanding values identified in Annex V (Article 3) of the Environmental Protocol, which recognises appropriate purposes for designating an ASPA (Table 1). These features came from all seven of the feature categories except tourism, where no features were recognised as relating to Annex V. The largest number of features related to classification (c) of Article 3: ‘areas with important or unusual assemblages of species, including major colonies of breeding native birds or mammals’.

## Discussion

The Antarctic Peninsula hosts a diverse range of terrestrial features of value. Many of these relate to the environmental, science, and intrinsic values traditionally recognised by the Antarctic Treaty and Environmental Protocol (Antarctic Treaty Secretariat 1991; Hemmings 2012). These reflect Antarctica’s remoteness and late discovery, lack of historical human occupation and indigenous peoples (Barr 2018), and the comparatively pristine landscapes (Watson et al. 2018). However, now thirty years after the Environmental Protocol was signed, it is clear that these traditional values do not reflect all features considered important by stakeholders today. This is particularly true of features identified as important to tourism. Resistance to tourism presence and growth, by many Antarctic Treaty Parties, may result in an unwillingness to recognise social values as important in Antarctica (Liggett et al. 2011; New Zealand 2017; Netherlands and New Zealand 2019). Yet, tourism is arguably the only way the global public can access Antarctic features and values. Recognising the importance of cultural and social considerations alongside environmental and ecological considerations is also increasingly appreciated by policymakers, managers and conservation practitioners around the world (Ban et al. 2013; Guerrero et al. 2018). Social-ecological systems have long been recognised as a foundational framework for equitable and effective conservation policy, giving voice to all stakeholders with an interest in a region (Ostrom 2009). Difficult conservation problems, including those now starting to emerge in the context of the combined effects of climate change, tourism and fishing in the Antarctic Peninsula region (Hogg et al. 2020), may benefit from applying social-ecological approaches. Whilst social-ecological frameworks have yet to be widely adopted in the region, they are now starting to be implemented as a basis for framing research to support policy (Hughes et al. 2022; Securing Antarctica’s Environmental Future 2021).

There is clear overlapping interest among stakeholders in many terrestrial features. Most prominently, all three stakeholders identified pygoscelid (Adélie, Gentoo, Chinstrap) and emperor penguin colonies, and fur and elephant seals, as important. This is not surprising given worldwide interest in charismatic species (Bennett et al. 2015; Sibarani et al. 2019), resulting in a great scientific interest in Antarctic vertebrates, particularly penguins, and Antarctic tourists’ strong desire to see these species (Bender et al. 2016; Lee 2019). Recognising the overlapping interest in these features will be crucial in developing and implementing achievable conservation actions that can cater to diverse stakeholder requirements, while meeting the objectives of the Antarctic Treaty and Environmental Protocol. The lack of interest from other stakeholders attached to many tourism features, such as opportunities for skiing or snorkelling, and sites providing a safe harbour for yachts, indicate that the tourism industry values some features that are of lower importance to science and biodiversity stakeholders. Some of these features may not overlap in space with important biodiversity and science features, such as areas suitable for skiing or snowshoeing, though others likely will (e.g. some sites suitable for conducting a ‘polar plunge’ from shore, like Deception Island, are also rich in biodiversity features). Several science features were only of interest to the science stakeholders, including sites of non-native species incursions, long-term monitoring sites and control sites, while all features identified as important to the biodiversity stakeholder group were also considered important to science stakeholders, who wish to better understand the biodiversity, habitat and geographic features in the Antarctic Peninsula. Science features were identified primarily by biologists, and although they considered the values likely to be appreciated by glaciologists, geologists and other Antarctic scientists, it is likely some features important to science have been overlooked.

Mounting recognition of global change and threats facing Antarctica have stimulated interest in further developing the Antarctic Specially Protected Area system (Coetzee et al. 2017; Argentina et al. 2018). Several studies have also highlighted the deficiencies of the current system (Shaw et al. 2014; Hughes and Grant 2017), which is not representative of all biodiversity groups (Wauchope et al. 2019; Phillips et al. 2022). Annex V of the Environmental Protocol identifies nine specific, although not exclusive, categories for designating an ASPA, and here we have identified forty-two features that fall into and represent these categories (Table 1). For example, barren soils, epi-shelf lakes and geothermal hotspots are examples of ‘areas with important or unusual assemblages of species, including major colonies of breeding native birds or mammals’. These forty-two features could be integrated into future ASPA planning work or considered in management plans. Consideration of social features, such as those identified as important for tourism, should also occur in alignment with a social-ecological approach to ASPA planning (Burrows et al. 2023), which is necessary for effective implementation of conservation plans (Bryan et al. 2011; Guerrero and Wilson 2017).

Although the precautionary principle should be applied, integration of features into conservation planning work hinges on data availability, which varies considerably. Most biodiversity features identified here have some data available, if only ad hoc and not comprehensive. Comprehensive data can provide a reliable representation of presence (and/or absence) of a feature across the landscape, i.e., a reasonably certain understanding of where the feature is located across an entire region. For example, the Mapping Application for Penguin Populations and Projected Dynamics (MAPPPD) project represents such data for Antarctic penguin colonies (Humphries et al. 2017; Che-Castaldo et al. 2023). Many of the features with ad hoc data available will not include all of the locations where that feature is present, only the ones known by the experts involved. Features with no spatial data available, such as ecological processes, or with only ad hoc data, such as unofficial historic sites, should be prioritised in future data collection or compilation to allow their integration into future conservation plans and management strategies.

Engaging with stakeholders is essential for successful conservation (Knight et al. 2008, 2010; Ban et al. 2013). To identify features of value in the Antarctic Peninsula for use in SCP we considered the most important stakeholders to be those that work directly in the Antarctic Peninsula region, with interests in biodiversity, science, and tourism. This includes scientists, policymakers, tourism operators, and NGOs, and every effort was made to engage with a diversity of experts within these fields (science and biodiversity experts came from 17 different Antarctic Treaty nations). However, there are a range of other stakeholders whose views were not directly incorporated, including tourists themselves, non-science staff who work at scientific stations, citizens of Antarctic gateway cities (particularly Ushuaia, Argentina and Punta Arenas, Chile), and the global public as a whole. Antarctic gateway cities are portals for goods and services to reach the Antarctic, but also have rich historical and contemporary connections to the continent (e.g. visits by heroic age explorers, sealing and whaling hubs, logistics centres for tourism or National Programmes; Roldan 2015; Leane et al. 2021). The Antarctic link generates work and income for gateway city citizens and some people develop connections with Antarctica, sometimes with a sense of custodianship (Roldan 2015; Leane et al. 2021). The global public frequently recognises Antarctica’s wilderness and aesthetic values, its role in driving global climate, and its vulnerability (McLean and Rock 2016; Tin et al. 2018; Nielsen 2019). Considering the views of a greater diversity of stakeholders could increase the number of features identified in the Antarctic Peninsula or change the proportion of stakeholders interested in features (the global public, for example, clearly values Antarctica’s ecosystem services). Although a diversity of participants were involved in the stakeholder engagement (countries, gender, and career stage), the research team come from Western nations, which may have also influenced the approach taken and framing of the project. This could have biased results towards western views and values, which should be recognised as a limitation of the approach.

## Conclusions

There are a diverse range of stakeholders with an interest in the Antarctic Peninsula, including scientists, tourist operators, citizens of gateway countries, the global public, and primarily the Antarctic Treaty Parties. These stakeholders’ all value biodiversity and want to see the best outcomes for the region’s natural environment, however, they also value a diversity of other features within the region. While the Antarctic Treaty and Environmental Protocol have traditionally emphasised science, environmental and intrinsic values, we suggest there is a need for an increased recognition of social values in Antarctica, especially those of tourism. This increased representation will lead to more realistic conservation and management. The 115 features of value identified in this work can be used to inform systematic conservation planning approaches, as well as other management or research endeavours. A primary example could be their consideration in site-based management, such as Visitor Site Guidelines for tourism, or Antarctic Specially Managed Areas (ASMAs), for sites with a multitude of users and values.

To provide impetus for these approaches, both the features of value identified here, and the benefits of the SCP approach more generally, using explicit examples from the Antarctic Peninsula, should be provided to the members of the CEP, encouraging them to further develop the work they commenced with the ATCM XLII CEP-SCAR Protected Area Planning Workshop (Argentina et al. 2018; Australia et al. 2019). By furthering dialogue with members of the CEP, broad stakeholder participation can develop approaches which might then be refined for presentation to the Antarctic Treaty Consultative Parties for consideration. Consensus decisions are not required by the CEP (Hughes et al. 2023), enabling a diversity of views to be considered.

Progression toward a common understanding is even more important given some of the difficulties in discussion recently encountered across the Antarctic Treaty System, for example agreement on the use of inviolate areas within ASPAs (Antarctic Treaty Secretariat 2023).

Such an approach might best be developed through existing collaborations, like the SCAR-IAATO systematic conservation planning project, in alignment with the substantial interest in developing the ASPA network demonstrated by the CEP.

### Supplementary Information

Below is the link to the electronic supplementary material.

Supplementary file1 (XLSX 21 KB)

Supplementary file2 (PDF 697 KB)

## References

[CR1] Adams VM, Mills M, Weeks R, Segan DB, Pressey RL, Gurney GG, Groves C, Davis FW (2019). Implementation strategies for systematic conservation planning. Ambio.

[CR2] Antarctic Treaty Secretariat. 1959. The Antarctic Treaty. 1st Dec 1959, Washington D.C.

[CR3] Antarctic Treaty Secretariat. 1966. Final Report of the Fourth Antarctic Treaty Consultative Meeting. pp. 2. Agenda Item 11. https://documents.ats.aq/ATCM4/fr/ATCM4_fr001_e.pdf.

[CR4] Antarctic Treaty Secretariat. 1991. Protocol on Environmental Protection to The Antarctic Treaty. 4th Oct 1991, Madrid, Spain.

[CR5] Antarctic Treaty Secretariat. 2017. Final Report of the Fortieth Antarctic Treaty Consultative Meeting. pp. 111. Para 416–417. https://documents.ats.aq/atcm40/fr/atcm40_fr001_e.pdf.

[CR6] Antarctic Treaty Secretariat. 2023. Final Report of the Forty-Fifth Antarctic Treaty Consultative Meeting. pp. 263. Decision 6. https://documents.ats.aq/ATCM45/fr/ATCM45_fr011_e.pdf.

[CR7] Argentina, Australia, Belgium, Chile, China, Czech Republic, France, Germany, et al. 2018. Proposal for a joint SCAR/CEP workshop on further developing the Antarctic protected area system. In: Working Paper 16. Antarctic Treaty Consultative Meeting XLI. 13–8 May 2018, Buenos Aires, Argentina.

[CR8] Argentina. 2022. Planning process for future capacity expansion of Petrel Base, Cape Welchness, Dundee Island. In: Information Paper 93. Antarctic Treaty Consultative Meeting XLIV. 23 May–2 June 2022, Berlin, Germany.

[CR9] Australia, Czech Republic, SCAR, and United States. 2019. Recommendations arising from the joint SCAR/CEP workshop on further developing the Antarctic protected area system, Prague, Czech Republic, 27–28 June 2019. In: Working Paper 70. Antarctic Treaty Consultative Meeting XLII. 2–11 July 2019, Prague, Czech Republic.

[CR10] Ban NC, Mills M, Tam J, Hicks CC, Klain S, Stoeckl N, Bottrill MC, Levine J (2013). A social–ecological approach to conservation planning: Embedding social considerations. Frontiers in Ecology and the Environment.

[CR11] Barr S (2018). Twenty years of protection of historic values in Antarctica under the Madrid Protocol. The Polar Journal.

[CR12] Bender NA, Crosbie K, Lynch HJ (2016). Patterns of tourism in the Antarctic Peninsula region: A 20-year analysis. Antarctic Science.

[CR13] Bennett JR, Maloney R, Possingham HP (2015). Biodiversity gains from efficient use of private sponsorship for flagship species conservation. Proceedings of the Royal Society B: Biological Sciences.

[CR14] Bryan BA, Raymond CM, Crossman ND, King D (2011). Comparing spatially explicit ecological and social values for natural areas to identify effective conservation strategies. Conservation Biology.

[CR15] Burrows JL, Lee JR, Wilson KA (2023). Evaluating the conservation impact of Antarctica's protected areas. Conservation Biology.

[CR16] Cajiao D, Albertos B, Tejedo P, Muñoz-Puelles L, Garilleti R, Lara F, Sancho LG, Tirira DG (2020). Assessing the conservation values and tourism threats in Barrientos Island, Antarctic Peninsula. Journal of Environmental Management.

[CR17] Carwardine J, Martin TG, Firn J, Reyes RP, Nicol S, Reeson A, Grantham HS, Stratford D (2019). Priority threat management for biodiversity conservation: A handbook. Journal of Applied Ecology.

[CR18] Che-Castaldo C, Humphries G, Lynch HJ (2023). Antarctic penguin biogeography project: Database of abundance and distribution for the Adélie, chinstrap, gentoo, emperor, macaroni and king penguin south of 60 S. Biodiversity Data Journal.

[CR19] Chown SL, Brooks CM (2019). The state and future of Antarctic environments in a global context. Annual Review of Environment and Resources.

[CR20] Chown SL, Clarke A, Fraser CI, Cary SC, Moon KL, McGeoch MA (2015). The changing form of Antarctic biodiversity. Nature.

[CR21] Coetzee BWT, Convey P, Chown SL (2017). Expanding the protected area network in Antarctica is urgent and readily achievable. Conservation Letters.

[CR22] COMNAP. 2023. COMNAP Antarctic facilities layer. https://github.com/PolarGeospatialCenter/comnap-antarctic-facilities. Accessed Mar 2023.

[CR23] Fretwell PT, Boutet A, Ratcliffe N (2023). Record low 2022 Antarctic sea ice led to catastrophic breeding failure of emperor penguins. Communications Earth & Environment.

[CR24] Gill P, Stewart K, Treasure E, Chadwick B (2008). Methods of data collection in qualitative research: Interviews and focus groups. British Dental Journal.

[CR25] Guerrero AM, Wilson KA (2017). Using a social-ecological framework to inform the implementation of conservation plans. Conservation Biology.

[CR26] Guerrero AM, Bennett NJ, Wilson KA, Carter N, Gill D, Mills M, Ives CD, Selinske MJ (2018). Achieving the promise of integration in social-ecological research: A review and prospectus. Ecology and Society.

[CR27] Haase D, Lamers M, Amelung B (2009). Heading into uncharted territory? Exploring the institutional robustness of self-regulation in the Antarctic tourism sector. Journal of Sustainable Tourism.

[CR28] Hemmings AD (2012). Considerable values in Antarctica. The Polar Journal.

[CR29] Hogg CJ, Lea M-A, Soler MG, Vasquez VN, Payo-Payo A, Parrott ML, Santos MM, Shaw J (2020). Protect the Antarctic Peninsula—Before it’s too late. Nature.

[CR30] Hughes KA, Grant SM (2017). The spatial distribution of Antarctica’s protected areas: A product of pragmatism, geopolitics or conservation need?. Environmental Science & Policy.

[CR31] Hughes KA, Pescott OL, Peyton J, Adriaens T, Cottier-Cook EJ, Key G, Rabitsch W, Tricarico E (2020). Invasive non-native species likely to threaten biodiversity and ecosystems in the Antarctic Peninsula region. Global Change Biology.

[CR32] Hughes KA, Santos M, Caccavo JA, Chignell SM, Gardiner NB, Gilbert N, Howkins A, Van Vuuren BJ (2022). Ant-ICON—‘Integrated Science to Inform Antarctic and Southern Ocean Conservation’: A new SCAR Scientific Research Programme. Antarctic Science.

[CR33] Hughes KA, Lowther A, Gilbert N, Waluda CM, Lee JR (2023). Communicating the best available science to inform Antarctic policy and management: A practical introduction for researchers. Antarctic Science.

[CR34] Humphries GRW, Naveen R, Schwaller M, Che-Castaldo C, McDowall P, Schrimpf M, Lynch HJ (2017). Mapping application for penguin populations and projected dynamics (MAPPPD): Data and tools for dynamic management and decision support. Polar Record.

[CR35] International Association of Antarctica Tour Operators. 2022. Report of the International Association of Antarctica Tour Operators 2021–22. In: Information Paper 41. Antarctic Treaty Consultative Meeting XLIV. 23 May–2 June 2022, Berlin, Germany.

[CR36] International Association of Antarctica Tour Operators. 2023. IAATO Overview of Antarctic Tourism: The 2022–23 season, and preliminary estimates for 2023–24. In: Information Paper 56. Antarctic Treaty Consultative Meeting XLV. 29 May–8 June 2023, Helsinki, Finland.

[CR37] Knight AT, Cowling RM, Rouget M, Balmford A, Lombard AT, Campbell BM (2008). Knowing but not doing: Selecting priority conservation areas and the research-implementation gap. Conservation Biology.

[CR38] Knight AT, Cowling RM, Difford M, Campbell BM (2010). Mapping human and social dimensions of conservation opportunity for the scheduling of conservation action on private land. Conservation Biology.

[CR39] Leane E, Lucas C, Marx K, Datta D, Nielsen H, Salazar JF (2021). From gateway to custodian city: Understanding urban residents’ sense of connectedness to Antarctica. Geographical Research.

[CR40] Lee JR, Raymond B, Bracegirdle TJ, Chadès I, Fuller RA, Shaw JD, Terauds A (2017). Climate change drives expansion of Antarctic ice-free habitat. Nature.

[CR41] Lee JR, Terauds A, Carwardine J, Shaw JD, Fuller RA, Possingham HP, Chown SL, Convey P (2022). Threat management priorities for conserving Antarctic biodiversity. PLOS Biology.

[CR42] Lee, J. R. 2019. Conserving Antarctic biodiversity in the Anthropocene. PhD Thesis. Brisbane, Australia: University of Queensland (Thesis)

[CR43] Liggett D, McIntosh A, Thompson A, Gilbert N, Storey B (2011). From frozen continent to tourism hotspot? Five decades of Antarctic tourism development and management, and a glimpse into the future. Tourism Management.

[CR44] Liggett D, Cajiao D, Lamers M, Leung Y-F, Stewart EJ (2023). The future of sustainable polar ship-based tourism. Cambridge Prisms: Coastal Futures.

[CR45] Lipps, J. H. 1978. Man's impact along the Antarctic Peninsula in Parker BC, and Holliman MC, editors. Environmental Impact in Antarctica. Blacksburg: Virginia Polytechnic Institute & State University.

[CR46] Margules CR, Pressey RL (2000). Systematic conservation planning. Nature.

[CR47] Martin TG, Burgman MA, Fidler F, Kuhnert PM, Low-Choy S, McBride M, Mengersen K (2012). Eliciting expert knowledge in conservation science. Conservation Biology.

[CR48] Maxwell SL, Cazalis V, Dudley N, Hoffmann M, Rodrigues ASL, Stolton S, Visconti P, Woodley S (2020). Area-based conservation in the twenty-first century. Nature.

[CR49] McLean L, Rock J (2016). The importance of Antarctica: Assessing the values ascribed to Antarctica by its researchers to aid effective climate change communication. The Polar Journal.

[CR50] Mukherjee N, Hugé J, Sutherland WJ, McNeill J, Van Opstal M, Dahdouh-Guebas F, Koedam N (2015). The Delphi technique in ecology and biological conservation: Applications and guidelines. Methods in Ecology and Evolution.

[CR51] Netherlands, and New Zealand. 2019. Proactive management of Antarctic tourism: Time for a fresh approach. In: Information Paper 26. Antarctic Treaty Consultative Meeting XLII. 2–11 July 2019, Prague, Czech Republic.

[CR52] Netherlands, and United Kingdom. 2019. Antarctic tourism workshop, 3–5 April in Rotterdam, The Netherlands: Chair’s summary and key recommendations. In: Working Paper 19. Antarctic Treaty Consultative Meeting XLII. 2–11 July 2019, Prague, Czech Republic.

[CR53] New Zealand. 2017. A strategic approach to environmentally managed tourism. In: Working Paper 31. Antarctic Treaty Consultative Meeting XL. 22 May–1 June 2017, Beijing, China.

[CR54] Nielsen H, Leane E, McGee J (2019). Save the penguins. Anthropocene Antarctica.

[CR55] Ostrom E (2009). A general framework for analyzing sustainability of social-ecological systems. Science.

[CR56] Phillips LM, Leihy RI, Chown SL (2022). Improving species-based area protection in Antarctica. Conservation Biology.

[CR57] R Core Team. 2022. R: A language and environment for statistical computing. R Foundation for Statistical Computing, Vienna, Austria. http://www.R-project.org.

[CR59] Reed MS (2008). Stakeholder participation for environmental management: A literature review. Biological Conservation.

[CR60] Robinson SA, Klekociuk AR, King DH, Pizarro RM, Zúñiga GE, Bergstrom DM (2020). The 2019/2020 summer of Antarctic heatwaves. Global Change Biology.

[CR61] Roldan G (2015). 'A door to the ice?: The significance of the Antarctic Gateway Cities today. Journal of Antarctic Affairs.

[CR62] Scientific Committee on Antarctic Research, and International Association of Antarctica Tour Operators. 2017. Systematic conservation plan for the Antarctic Peninsula. In: Information Paper 166. Antarctic Treaty Consultative Meeting XL. 22 May–1 June 2017, Beijing, China.

[CR63] Scientific Committee on Antarctic Research, and International Association of Antarctica Tour Operators. 2023. Systematic conservation plan for the Antarctic Peninsula project updates and next steps. In: Information Paper 48. Antarctic Treaty Consultative Meeting XLV. 29 May–8 June 2023, Helsinki, Finland.

[CR64] Securing Antarctica’s Environmental Future. 2021. Annual Report. https://arcsaef.com/annual-reports/. Accessed June 2023.

[CR65] Shaw JD, Terauds A, Riddle MJ, Possingham HP, Chown SL (2014). Antarctica’s protected areas are inadequate, unrepresentative, and at risk. PLoS Biology.

[CR66] Sibarani MC, Di Marco M, Rondinini C, Kark S (2019). Measuring the surrogacy potential of charismatic megafauna species across taxonomic, phylogenetic and functional diversity on a megadiverse island. Journal of Applied Ecology.

[CR67] Siegert M, Atkinson A, Banwell A, Brandon M, Convey P, Davies B, Downie R, Edwards T (2019). The Antarctic Peninsula under a 1.5 °C global warming scenario. Frontiers in Environmental Science.

[CR68] Slater T, Shepherd A (2018). Antarctic ice losses tracking high. Nature Climate Change.

[CR69] Slocum N (2005). Participatory Methods Toolkit—A Practitioner’s Manual.

[CR70] Summerson R, Tin T (2018). Twenty years of protection of wilderness values in Antarctica. The Polar Journal.

[CR71] Tejedo P, Pertierra LR, Benayas J, Convey P, Justel A, Quesada A (2012). Trampling on maritime Antarctica: Can soil ecosystems be effectively protected through existing codes of conduct?. Polar Research.

[CR72] Tejedo P, Benayas J, Cajiao D, Leung Y-F, De Filippo D, Liggett D (2022). What are the real environmental impacts of Antarctic tourism? Unveiling their importance through a comprehensive meta-analysis. Journal of Environmental Management.

[CR73] Tin T, O’Reilly J, Peden J, Pinkalla S, Kelly M, Larrea K, Haugen B, Haun A (2018). Perceptions of wilderness and the Antarctic: Case studies from the United States. The Polar Journal.

[CR74] Walton DWH (2018). Tourism in the Antarctic. Antarctic Science.

[CR75] Watson JEM, Venter O, Lee JR, Jones KR, Robinson JG, Possingham HP, Allan JR (2018). Protect the last of the wild. Nature.

[CR76] Wauchope HS, Shaw JD, Terauds A (2019). A snapshot of biodiversity protection in Antarctica. Nature Communications.

[CR77] Whitehead AL, Kujala H, Ives CD, Gordon A, Lentini PE, Wintle BA, Nicholson E, Raymond CM (2014). Integrating biological and social values when prioritizing places for biodiversity conservation. Conservation Biology.

[CR78] Wouters B, Martin-Español A, Helm V, Flament T, van Wessem JM, Ligtenberg SRM, van den Broeke MR, Bamber JL (2015). Dynamic thinning of glaciers on the Southern Antarctic Peninsula. Science.

