# Supplementary material for: Conservation features of the terrestrial Antarctic Peninsula
Source: Ambio. 2024 Apr 8;53(7):1037–49. doi: 10.1007/s13280-024-02009-4 (PMC11101391; doi:10.1007/s13280-024-02009-4)

Ambio

Supplementary Information
This supplementary information has not been peer reviewed.

Title: **Conservation features of the terrestrial Antarctic Peninsula**

Table S1. The 115 conservation features identified in the Antarctic Peninsula by three stakeholder groups. Each of the features is divided into categories and subcategories and includes information on which of the three stakeholders value the feature, and whether or not there is spatial data available. The three stakeholders include biodiversity, science, and tourism.

Table S1 is available as an excel spreadsheet.

Table S2. Summary of 115 identified conservation features of value in the Antarctic Peninsula, divided into seven broad categories and narrower subcategories. The *'Number of features'* represents the total number of features identified in each subcategory. *'Example'* gives an example of one feature in the subcategory and *'Data availability'* gives the percentage of features that have comprehensive data available. *'Stakeholders'* represent the stakeholders who have identified features under each subcategory, where B = biodiversity, S = science, and T = tourism. All features are listed in in Table S1.

Category	Subcategory	Number of features	Example	Data availability*	Stakeholders
Biodiversity	Invertebrates	15	Free-living mites	93%	B + S + T
	Microbes	9	Dry soil microbial communities	78%	B + S
	Seabirds	14	Adélie penguins	93%	B + S + T
	Seals	3	Antarctic fur seals	67%	B + S + T
	Vegetation	12	Bank-forming mosses	100%	B + S + T
	Whales**	3	Killer whales	0%	T
Habitats	General habitats	4	Rock substrate	50%	B + S
	Unique assemblages	10	Geothermal/volcanic sites	20%	B + S
Geographic	General geographic	5	Ecological processes	40%	B + S
	Geological	4	Fossil bearing rocks	0%	S + T
	Ice	3	Glaciers	67%	S + T
	Location	2	Continental landing	100%	T
Historic	Historic	2	Historic sites and monuments (HSM's)	50%	S + T
Science	Infrastructure	4	Scientific research facilities	100%	S + T
	Non-native species	1	Sites of non-native species incursions	100%	S
	Science	6	Important long-term monitoring sites	17%	S + T
Intrinsic	Intrinsic	4	Wilderness	50%	S + T
Tourism	Accessibility	4	Safe harbour for yachts	0%	T
	Activities	10	Camping	0%	T

**Percentages do not include ad hoc data which is available for some features.*
***Whales are included on this list as a tourism feature in a terrestrial sense, where expeditions may make landings at a site in order to do a split landing/zodiac cruise where whales are associated with the site.*

Figure S1. Flow chart detailing the six steps of the stakeholder engagement process for deriving features of value for the terrestrial Antarctic Peninsula.

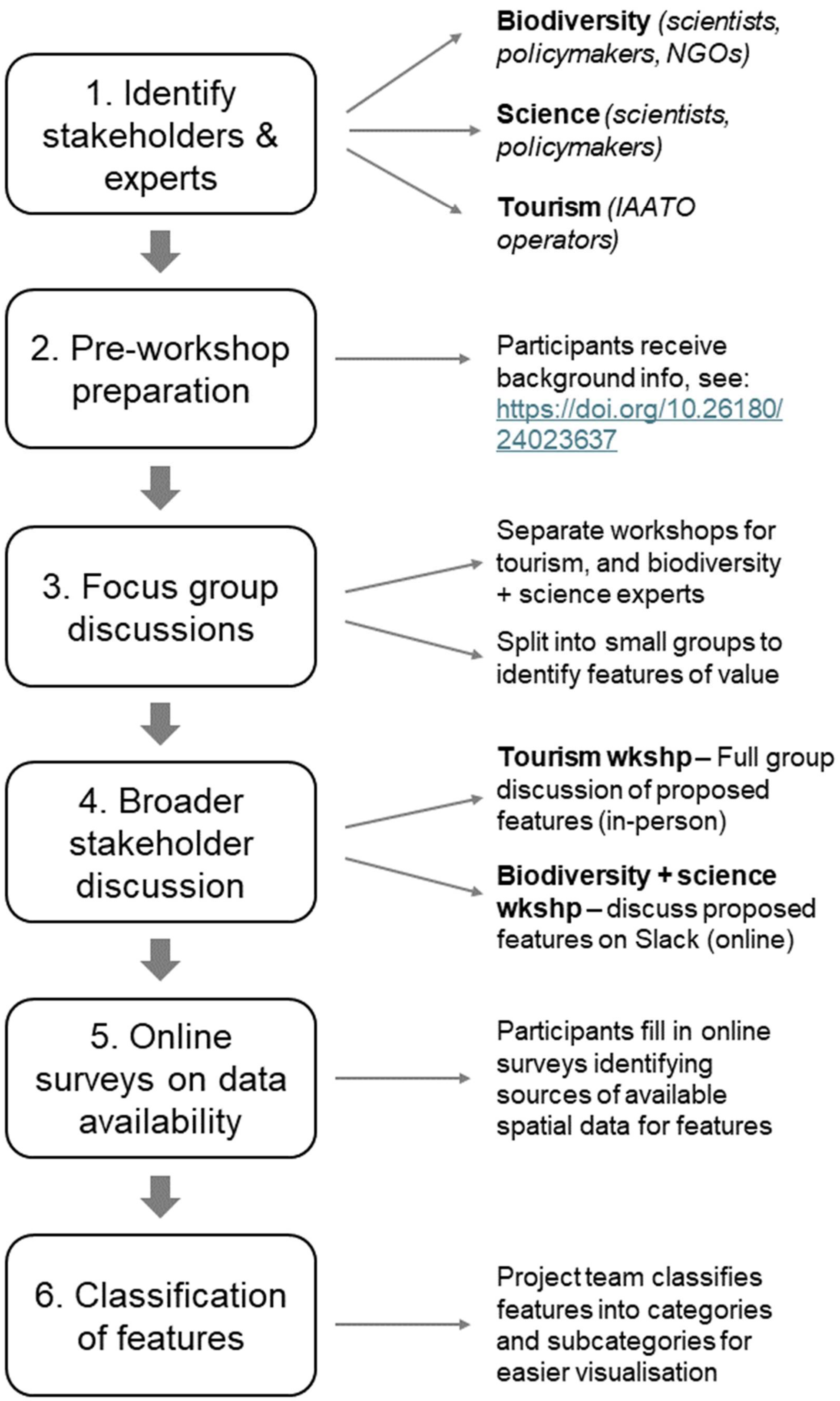

Supplement: Supplementary file 2 — Supplementary file2 (PDF 697 KB) [file 13280_2024_2009_MOESM2_ESM.pdf]
